# Integration of CD34^+^CD117^dim^ population signature improves the prognosis prediction of acute myeloid leukemia

**DOI:** 10.1186/s12967-022-03556-8

**Published:** 2022-08-12

**Authors:** Xue-Ping Li, Wei-Na Zhang, Jia-Ying Mao, Bai-Tian Zhao, Lu Jiang, Yan Gao

**Affiliations:** 1 Department of Hematologic Oncology, Sun Yat-sen University Cancer Center, Guangzhou, 500020 China; 2 State Key Laboratory of Oncology in South China, Collaborative Innovation Center for Cancer Medicine, 651 Dongfeng East Road, Guangzhou, 500020 China; 3 Department of Hematology, Guangzhou Women and Children’s Medical Center, Guangzhou Medical University, Guangzhou, 500020 China; 4 Department of Medical Oncology, Sun Yat-sen University Cancer Center, Guangzhou, China; 5 Shanghai Institute of Hematology, State Key Laboratory of Medical Genomics, National Research Center for Translational Medicine at Shanghai, Ruijin Hospital, Shanghai Jiao Tong University School of Medicine, Shanghai, 200025 China

**Keywords:** Acute myeloid leukemia, Gene expression profile, ELN 2017, Prediction model

## Abstract

**Background:**

Acute Myeloid Leukemia (AML) is a hematological cancer characterized by heterogeneous hematopoietic cells. Through the use of multidimensional sequencing technologies, we previously identified a distinct myeloblast population, CD34^+^CD117^dim^, the proportion of which was strongly associated with the clinical outcome in t (8;21) AML. In this study, we explored the potential value of the CD34^+^CD117^dim^ population signature (117DPS) in AML stratification.

**Methods:**

Based on the CD34^+^CD117^dim^ gene signature, the least absolute shrinkage and selection operator (LASSO) Cox regression analysis was performed to construct the 117DPS model using the gene expression data from Gene Expression Omnibus (GEO) database (GSE37642-GPL96 was used as training cohort; GSE37642-GPL570, GSE12417-GPL96, GSE12417-GPL570 and GSE106291 were used as validation cohorts). In addition, the RNA-seq data from The Cancer Genome Atlas (TCGA)-LAML and Beat AML projects of de-novo AML patients were also analyzed as validation cohorts. The differences of clinical features and tumor-infiltrating lymphocytes were further explored between the high-risk score group and low-risk score group.

**Results:**

The high-risk group of the 117DPS model exhibited worse overall survival than the low-risk group in both training and validation cohorts. Immune signaling pathways were significantly activated in the high-risk group. Patients with high-risk score had a distinct pattern of infiltrating immune cells, which were closely related to clinical outcome.

**Conclusion:**

The 117DPS model established in our study may serve as a potentially valuable tool for predicting clinical outcome of patients with AML.

**Supplementary Information:**

The online version contains supplementary material available at 10.1186/s12967-022-03556-8.

## Background

Acute myeloid leukemia (AML) is a heterogeneous hematological cancer that arises from the clonal proliferation of malignant myeloid precursor cells and exhibits rapid progression [1]. The European Leukemia Net (ELN) for AML risk stratification 2017 [2] has been clinically adopted as a standard guideline. However, AML patients still faces major challenges related to drug resistance and relapse. An in-depth study of the prognostic clinical factors of AML will help improve the prognostic stratification and treatment efficacy.

Our previous studies [3, 4] identified the heterogeneous CD34^+^CD117^dim^ and CD34^+^CD117^bright (bri)^ myeloblast populations in patients with t (8;21) AML. These myeloblasts are blocked at different stages of myeloid differentiation and have distinct molecular and clinical characteristics that identified through several approaches, including RNA sequencing (RNA-seq), single-cell RNA-seq, and morphological and immuno-phenotypic analyses. The CD34^+^CD117^dim^ myeloblasts are found to be present at the earliest myeloid stage, exhibit high expression levels of granulocyte-monocyte progenitor markers, present a leukemia stem cell gene signature, and are drug-resistant to chemotherapy. scRNA-seq results at different disease time points identified CD34^+^CD117^dim^ myeloblasts as an important leukemic population which expanded at refractory stage after several cycles of chemotherapy. Univariate and multivariate analyses identified the proportion of CD34^+^CD117^dim^ myeloblasts as an independent factor for clinical outcome in AML. Patients with a higher CD34^+^CD117^dim^ proportion exhibited a poorer overall survival (OS). Further studies indicated that patients with higher expression levels of CD34^+^CD117^dim^-associated genes experienced an inferior OS [4]. Therefore, CD34^+^CD117^dim^ population is a group of myeloblasts with a high degree of malignancy, the proportion of which is significantly associated with prognosis. Establishing a prognostic model based on the signature gene-set of CD34^+^CD117^dim^ population and using this model as one of the risk factors may help improving the capability for risk prediction and prognosis prediction of AML. In this study, we aimed to investigate the potential prognostic value of the CD34^+^CD117^dim^ population signature (117DPS) model for clinical risk stratification system in AML patients.

## Methods

### Study population

RNA-seq data from 62 t (8;21) AML patients in our previous study were deposited at the National Omics Data Encyclopedia (NODE) (http://www.biosino.org/node/project/detail/OEP000629) and detailed treatment information was provided as previously described [3]. GEO: GSE37642 (AMLCG1999), GSE12417 and GSE106291 (AMLCG2008) could be downloaded from the Gene Expression Omnibus (GEO) databases. RNA-seq data from the Beat AML project [5] could be accessed by following the authors’ instructions. The Beat AML cohort was composed of 562 patients diagnosed with primary and relapse AML. However, only de novo AML cases with available survival information (*n* = 200) were selected for the subsequent analysis. RNA-seq data of TCGA-LAML cohort were downloaded from the online database (https://portal.gdc.cancer.gov/) [6].

### Construction of the CD34^+^CD117^dim^ gene signature

Differentially expressed genes (DEGs) between the CD34^+^CD117^dim^%-high group and the CD34^+^CD117^dim^%-low group obtained from the RNA-seq data of 62 t (8;21) AML patients in our previous study were identified as previously described [3]. Overexpressed genes of the CD34^+^CD117^dim^%-high group were determined with a *P* Value < 0.001 and an average log fold change (avg_logFC) > 1.0 [4]. The least absolute shrinkage and selection operator (LASSO) Cox regression analysis [7] was then applied to the training cohort (GSE37642-GPL96) to construct the CD34^+^CD117^dim^ population signature (DPS). To choose the optimal value for the λ parameter with the minimum criteria, parameters “family = ‘cox’, maxit = 1000” were used. Finally, with the λ value, we obtained the 117DPS model consisting of six genes and model coefficients. The overall survival analysis was conducted using the Kaplan–Meier analysis and the *P* value were compared using the log-rank test. For specificity and sensitivity analysis, ROC curve analysis using the timeROC package [8] was performed to evaluate the area under the curve (AUC) values.

### Protein–protein interactions (PPI) network construction

STRING database (https://cn.string-db.org) was used to predict protein–protein interactions (PPI) network of the overexpressed genes identified from CD34^+^CD117^dim^ population [9]. The minimum required interaction score was 0.4. The processed interaction predictions included text-mining, experiments, databases, et al.

### Immune infiltration analysis

CIBERSORT was used to investigate the enrichment of immune cells in the bone marrow microenvironment of AML patients [10]. For each sample, a relative abundance of 22 types of infiltrating immune cells, including T cells, B cells, NK cells, macrophages were analyzed. Correlation between immune cells inferred by CIBERSORT and 117DPS model was evaluated by Spearman correlation. Distribution of immune cells between high- and low-risk groups was compared using two-sided Wilcoxon test *P*-values.

### Gene set enrichment analysis

Gene set enrichment analysis (GSEA) was performed by GSEA software (http://software.broadinstitute.org/gsea/login.jsp) [11]. HALLMARK gene sets (H) and MSigDB curated gene sets (C2) were used.

### Statistical analysis

The comparison of the clinical characteristics between the high- and low-risk groups were performed with SPSS 22.0 (IBM). For categorical parameters, the χ2-test or Fisher’s exact test was used, while the Mann–Whitney U test was used for continuous variable. Univariate and multivariate Cox regression analyses were performed for the contributions of clinical factors to overall survival (OS) in AML patients. The other statistical analyses were performed using the R software (version 4.0.2, https://www.r-project.org/).

### Ethics statement

This study was approved by the Ruijin Hospital Review Board and informed consent was obtained from all patients in accordance with the Declaration of Helsinki.

## Results

### Identification of the over-expressed genes of the CD34^+^CD117^dim^ population

The differentially expressed genes (DEGs) between the CD34^+^CD117^dim^%-high group and the CD34^+^CD117^dim^%-low group were extracted from the RNA-seq data of 62 t (8;21) AML patients from our previous study [3]. To identify the gene-set of the CD34^+^CD117^dim^ population, genes of average log fold change (avg_logFC) > 1.0 and *P* value < 0.001 in the CD34^+^CD117^dim^%-high group were selected. Thus, we obtained a 45-gene set (Additional file 8: Table S1) with little interaction among them through protein–protein interaction (PPI) network (Additional file 1: Fig. S1).

### Establishment of the CD34^+^CD117^dim^ population signature (117DPS)

**Fig. 1:**
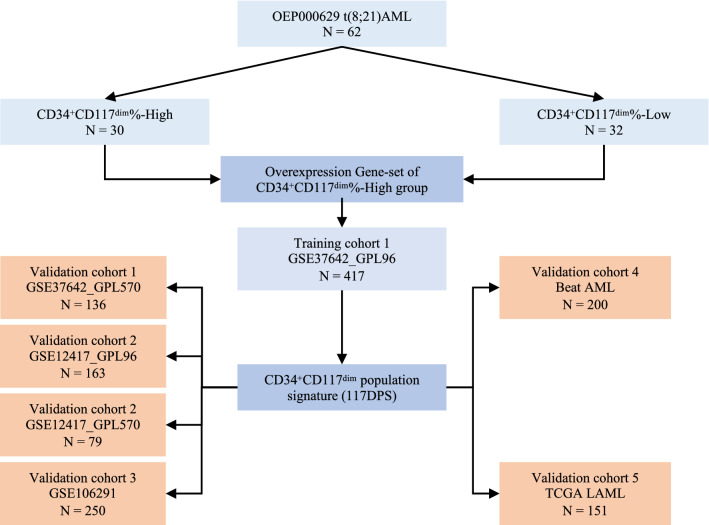
Flow chart showing the study design of the estimation and validation of the 117DPS model

Next, public AML datasets with clinical data available were used for the 117DPS model estimation and validation. Gene expression data from five GEO datasets including GSE37642 (GPL96, *n* = 417), GSE37642 (GPL570, *n* = 136), GSE12417 (GPL96, *n* = 163), GSE12417 (GPL570, *n* = 79) and GSE106291 (*n* = 250) were analyzed. In addition, RNA-seq data from TCGA including LAML (*n* = 151) and Beat AML (*n* = 200) of de-novo AML patients were also included in this study. GSE37642-GPL96 was used as the training cohort and the rest six datasets served as validation cohorts. A total of 1396 AML patients with clinical data available were analyzed in this study and the whole design was summarized in Fig. 1.

**Fig. 2:**
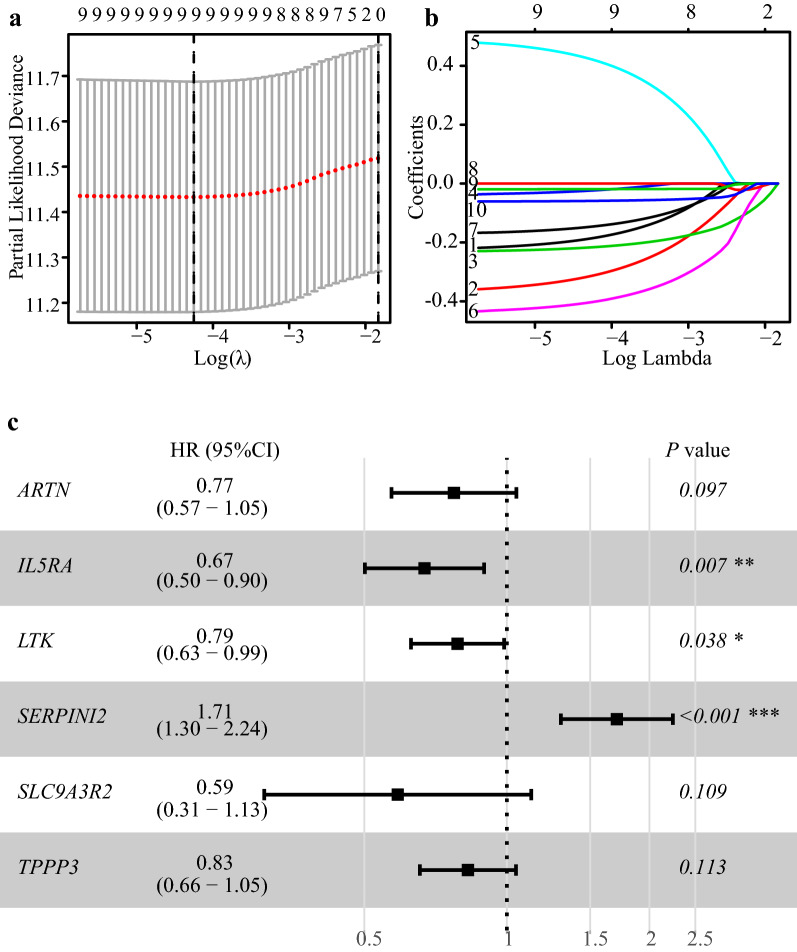
Development of the CD34^+^CD117^dim^ population signature (117DPS model). **a** One thousand bootstrap replicates were obtained by LASSO regression analysis for variable selection. The optimal value for the λ parameter was determined by the minimum criteria and 1-SE criteria. **b** LASSO coefficients of the CD34^+^CD117^dim^-related genes in the training cohort (GEO: GSE37642-GPL96). Each curve represents one gene. **c** Forest plot of a multivariate analysis of the six genes included in the 117DPS model using the GSE37642-GPL96 dataset. *HR* hazard ratio; *CI* confidence interval. *P < 0.05; **P < 0.01; ***P < 0.001

In the training cohort (GSE37642-GPL96), through univariate Cox regression analysis, ten out of the 45-gene set including *ARTN*, *IL5RA*, *LTK*, *MYRF*, *SERPINI2*, *SLC9A3R2*, *TPPP3*, *TPSAB1*, *TPSB2* and *TUBB3*, were found to be associated with the OS of patients with AML (Additional file 2: Fig. S2). Only *SERPINI2* was associated with inferior survival of patients with AML, and the other nine genes were correlated with better prognosis. Subsequently, to identify the optimal weighting coefficients, a least absolute shrinkage and selection operator (LASSO) regression analysis was performed using a penalized maximum likelihood estimator with 1000 bootstrap replicates (Fig. 2a and b) to derive a six-gene risk model. The risk score for the 117DPS model was calculated using the following formula: 117DPS = (− 0.257) × *ARTN* expression + (− 0.400) × *IL5RA* expression + (− 0.239) × *LTK* expression + 0.534 × *SERPINI2* expression + (− 0.530) × *SLC9A3R2* expression + (− 0.189) × *TPPP3* expression (Fig. 2c and Additional file 3: Fig. S3).

**Fig. 3:**
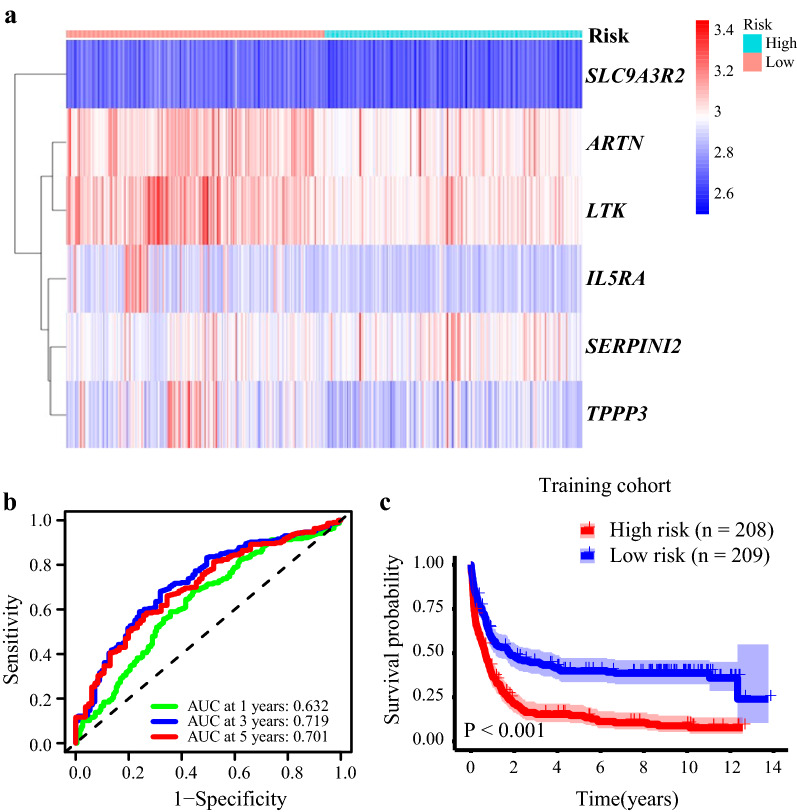
ROC curves and survival analysis of the 117DPS model in the training cohort. **a** The expression patterns of the six genes in patients with different risk scores. **b** Sensitivity and specificity of the 117DPS model by a receiver operating characteristic (ROC) analysis using the GSE37642-GPL96 dataset. AUC (area under the curve). **c** Survival differences between the high- and low-risk groups classified based on the 117DPS score in the GSE37642-GPL96 dataset. The log-rank *P* value is indicated

The expression patterns of the six genes differed in patients with different risk scores (Fig. 3a). In order to explore the prognostic accuracy of the 117DPS model in the training cohort, we performed a time-dependent receiver operating characteristic (ROC) analysis. In the training cohort (GSE37642-GPL96), the area under the curve (AUC) values of 1-, 3- and 5-year OS were 0.632, 0.719 and 0.701, respectively (Fig. 3b). Next, the OS of the high- and low-risk groups classified based on the median value of the 117DPS model (cut-off = 1.0514) was compared using the log-rank test. The Kaplan–Meier plot showed that the high-risk group (*n* = 208) had a significantly shorter OS (*P* < 0.001) (Fig. 3c). The five-year survival for the high- and low-risk group was 14.63% (10.38–20.63%) and 39.81% (33.58–47.19%), respectively.

### Validation of the 117DPS signature in external GEO cohorts

**Fig. 4:**
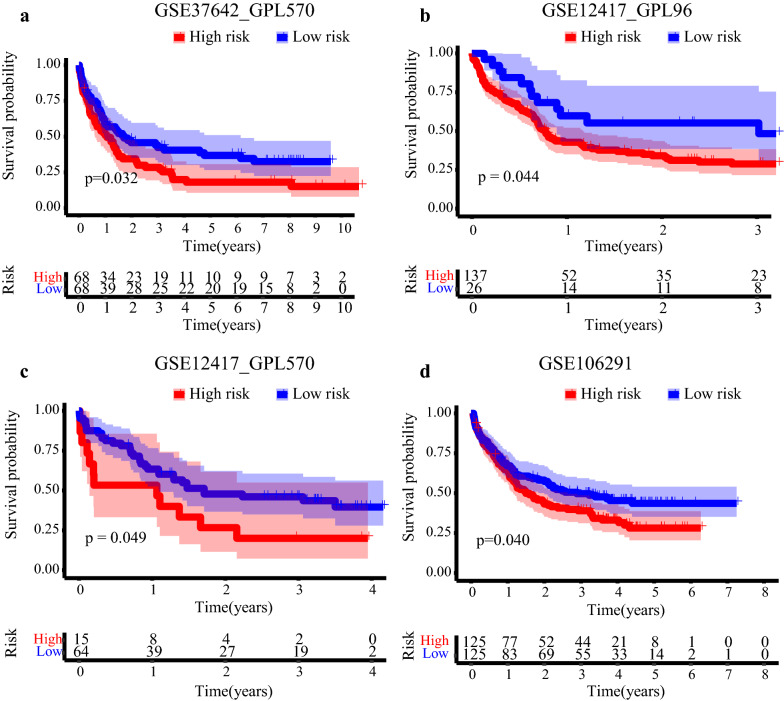
Validation of the 117DPS model in external GEO cohorts. **a** Survival differences between the high- and low-risk groups classified based on the 117DPS score in the GSE37642-GPL570 dataset. The log-rank *P* value is indicated. **b** Survival differences between the high- and low-risk groups classified based on the 117DPS score in the GSE12417-GPL96 dataset. The log-rank *P* value is indicated. **c** Survival differences between the high- and low-risk groups classified based on the 117DPS score in the GSE12417-GPL570 dataset. The log-rank *P* value is indicated. **d** Survival differences between the high- and low-risk groups classified based on the 117DPS score in the GSE106291 dataset. The log-rank *P* value is indicated

Further, the prognostic value of the 117DPS model was estimated in the validation cohorts. After classifying patients into high- and low-level groups based on the optimal cutoff value of the 117DPS model, we observed that the high-risk groups exhibited worse OS as in GSE37642_GPL570 (*P* = 0.032), GSE12417_GPL96 (*P* = 0.044), GSE12417_GPL570 (*P* = 0.049) and GSE106291 (*P* = 0.040) (Fig. 4). The 5-year survivals for the high- and low-risk group in GSE37642_GPL570 were 18.12% (10.68–30.74%) and 36.85% (26.61–51.02%), respectively (Fig. 4a). In GSE12417_GPL96, the three-year survival for the high- and low-risk group were 28.80% (21.60–38.30%) and 55.20% (38.50–79.10%), respectively (Fig. 4b). In GSE12417_GPL570, the three-year survival for the high- and low-risk group were 20.00% (7.27–55.03%) and 46.02% (34.96–60.57%), respectively (Fig. 4c). In GSE106291, the three-year survival for the high- and low-risk group were 38.95% (31.14–48.71%) and 49.67% (41.55–59.39%), respectively (Fig. 4d). The AUC values in GSE37642_GPL570, GSE12417_GPL96, GSE12417_GPL570 and GSE106291 were 0.632, 0.637, 0.646, and 0.612 (Additional file 4: Fig. S4), respectively.

### Validation of the 117DPS signature in TCGA and Beat AML cohorts

**Fig. 5:**
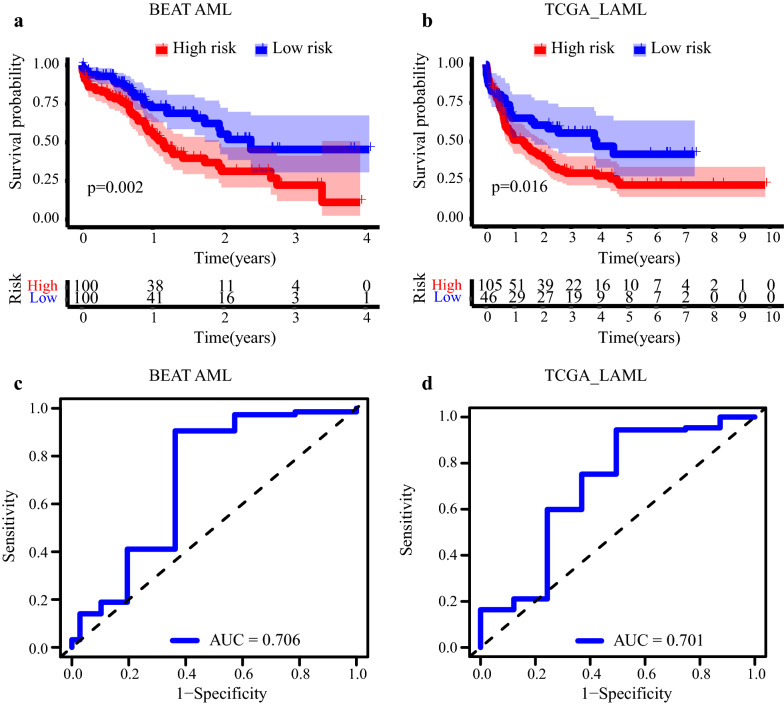
Validation of the 117DPS model in the TCGA LAML and Beat AML cohorts. **a** Kaplan–Meier plot of OS of the high- and low-risk groups classified based on the median cut-off value of 117DPS score in the Beat AML cohort (*n* = 200) with the indicated log-rank *P* value. **b** Kaplan–Meier plot of OS of the high- and low-risk groups classified based on the median cut-off value of 117DPS score in the TCGA AML cohort (*n* = 151) with the indicated log-rank *P* value. **c** Sensitivity and specificity of the 117DPS model by a receiver operating characteristic (ROC) analysis in the Beat AML cohort. **d** Sensitivity and specificity of the DPS model by a receiver operating characteristic (ROC) analysis in the TCGA AML cohort

Thus, the prognostic value of 117DPS model from microarray platform was verified and we wondered whether the performance of 117DPS model in RNA-seq data would be satisfying. The RNA-seq data from Beat AML [5] cohort included 562 patients that were diagnosed with primary and relapse stages. Nevertheless, only the de-novo AML patients with survival information available (*n* = 200) were selected for the subsequent analysis. Patients were classified into the high- and low-level groups with the median cut-off value (= 22.71) of 117DPS model. The Kaplan–Meier plot showed that the high-risk group (*n* = 100) had a significantly shorter OS (*P* = 0.002) (Fig. 5a). The 3-year survival for the high- and low-risk group were 22.30% (12.00–41.44%) and 45.38% (30.55–67.42%), respectively. For the RNA-seq dataset of TCGA-AML, the same cut-off value with Beat AML was adopted. The Kaplan Meier survival also demonstrated patients of high-risk group (*n* = 105) presented an inferior outcome (*P* = 0.016) (Fig. 5b). The three-year survival for the high- and low-risk group were 21.90% (14.20–33.60%) and 41.78% (27.44–63.61%), respectively. The AUC values of three-year OS in Beat AML and TCGA AML were 0.706 and 0.701, respectively (Fig. 5c and d).

### The associations between 117DPS signature and clinical characteristics in AML patients

In addition, we analyzed the distribution of clinical characteristics, including age, gender and other information, based on the information available from each database, between the high- and low-risk groups from the 117DPS model in the training and validation cohorts. In the training cohort, the median age of the high-risk group was 63 years, which was significantly higher than that of patients in the low-risk group (52 years) (*P* < 0.001). This was also validated in the validation cohorts (Additional file 9: Tables S2–S7). The patients of high-risk group harbored more *RUNX1* mutations and less *RUNX1-RUNX1T1* fusions compared with those of low-risk group as revealed by data from GSE37642 (Additional file 9: Tables S2 and S3). In addition, the high-risk group had a greater proportion of patients classified as intermediate/high risk based on ELN2017 stratification in Beat AML and TCGA LAML cohorts (Additional file 9: Tables S6 and S7).

### Improvement of the ELN2017 risk system in the prognosis prediction of AML patients

**Fig. 6:**
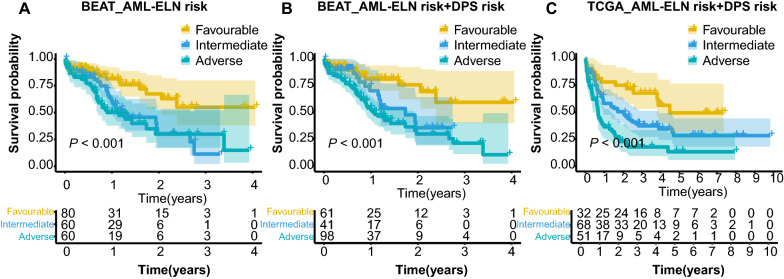
Incorporation with the ELN2017 risk system for AML stratification. **a** Kaplan–Meier plot of OS of the risk groups of the Beat AML cohort (*n* = 200) classified based on the ELN 2017 risk stratification system with the indicated log-rank *P* value. **b** Kaplan–Meier plot of OS of the risk groups classified based on the ELN 2017 risk stratification system of the Beat AML cohort (*n* = 200) classified based on the ELN 2017 risk stratification system plus DPS model with the indicated log-rank *P* value. **c** Kaplan–Meier plot of OS of the risk groups of the TCGA cohort (*n* = 151) classified based on the ELN 2017 risk stratification system plus DPS model with the indicated log-rank *P* value

Furthermore, we investigated whether the addition of 117DPS model in the ELN2017 system could achieve a better stratification for AML patients. In Beat AML cohort, the Kaplan–Meier analysis of ELN-117DPS model presented an improved stratification (Fig. 6a and b). In addition, the ELN-117DPS model for TCGA-AML cohort also showed a better stratification (Fig. 6c). Accordingly, ELN plus 117DPS model could more accurately define the clinical outcome for AML patients.

### Immune dysregulation in the high-risk group of 117DPS model

**Fig. 7:**
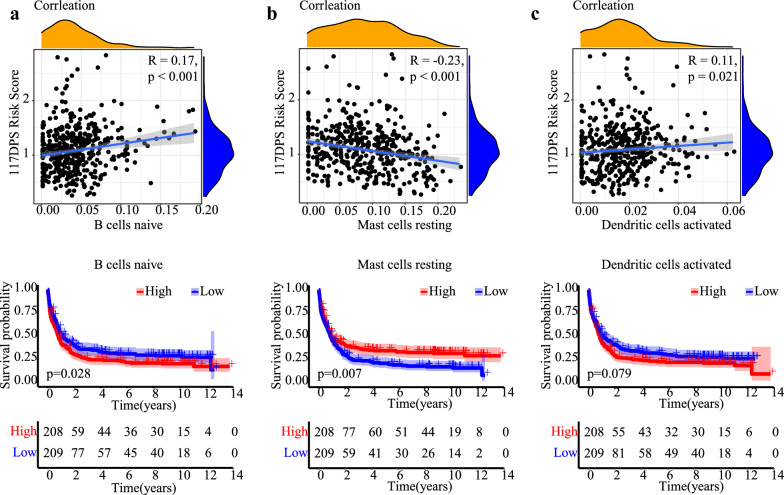
Infiltrated immune cells correlated with 117DPS model in training cohort. Correlation analysis (left) and Kaplan–Meier plot (right) of B cells naive (**a**), resting mast cells (**b**) and activated dendritic cells (**c**). R indicates correlation, and the correlation was evaluated by Spearman correlation. Survival differences was compared using the log-rank test

The proposed 117DPS model is based on the six genes including *ARTN*, *IL5RA*, *LTK*, *SERPINI2*, *SLC9A3R2* and *TPPP3*, all of which are known to participate in immune system and inflammation. To further clarify the molecular mechanism underlying the 117DPS model, we conducted a gene set enrichment analysis (GSEA) for the differentially expressed genes between high- and low- risk groups in the training cohort. The results revealed significant activation of immune signaling pathways (Additional file 5: Fig. S5a), including the interferon-gamma response and interferon-alpha response, in the high-risk group. In contrast, the low-risk group exhibited significant activation of the glycolysis and oxidative phosphorylation pathways (Additional file 5: Fig. S5b). Thus, this aberrant activation of immune signaling indicated that the immune mechanism might play an important role in the pathogenesis of the high-risk group in AML. Then, we calculated the proportions of 22 types of immune cells in each AML sample and compared the differences in proportions of immune cells between the high- and low-risk score groups using the CIBERSORT algorithm (Additional file 6: Fig. S6). We further performed a correlation analysis between immune cells and the 117DPS risk score. The 117DPS model was negatively correlated with resting mast cells (*R* = − 0.23, *P* < 0.001) (Fig. 7b). The 117DPS model was positively correlated with B cells naive (*R* = 0.17, *P* < 0.001) and activated dendritic cells (*R* = 0.11, *P* = 0.021) (Fig. 7a and c), which were closely related to clinical outcome.

In addition, we compared the expression levels of immune checkpoints and their ligands between the high- and low- risk score group. Patients with high-risk scores had a significantly higher expression of *LAG3* and *PDCD1* compared with those with low-risk score (Additional file 7: Fig. S7).

## Discussion

The clinical outcome of acute myeloid leukemia patients could be divergent and calls for more precise and improved risk stratification system. Currently, genetic and clinical factors, including cytogenetic and mutational events, are widely used in clinical practice. Recently, the updated European Leukemia Net (ELN) guidelines [2] incorporated gene mutations for AML stratification and Papaemmanuil et al. [12] proposed 14 subtypes of acute myeloid leukemia according to the genetic heterogeneity. Nonetheless, nearly 50% of patients are stratified into an intermediate-risk group [1] and remain obscure for the appropriate therapy regimen.

In this study, we constructed the CD34^+^CD117^dim^ population signature based on our previous findings in AML patients with t (8;21) [3, 4] and further explored its prognostic value in the whole AML cohort. The ratio of CD34^+^CD117^dim^ population, first identified in the t (8;21) AML subtype, were proved to be associated with the disease clinical outcome and were efficient in stratifying the patients when combined with *KIT* mutations in t (8;21) AML. When employed the 117DPS model in AML patients, both the training cohort and the validation cohort demonstrated a good performance of stratification power. Notably, though the data of training cohort was based on the gene expression profile from microarray platform, the validation were performed in cohorts including the RNA-sequencing data from two independent cohorts. Although the AUC values in the validation cohorts were not as satisfying as those in the training cohort, the Kaplan–Meier survival analysis of the 117DPS model still exhibited value for risk stratification. In addition, when incorporated with the ELN2017 risk system, the 117DPS model could be utilized in the intermediate-risk group of AML patients and provided potential for clinical application.

The six genes in this CD34^+^CD117^dim^ population signature model are *ARTN*, *IL5RA*, *LTK*, *SERPINI2*, *SLC9A3R2* and *TPPP3*, all of which have been known to play roles in immune response and gene regulation [13–19]. Recently, numerous studies have shown that a subset of AML patients may benefit from the immunotherapy. Our analysis revealed that there was significant activation of immune signal pathways such as IL6-JAK-STAT3 and interferon-gamma-response in the high-risk group of 117DPS model. In addition, high-risk group of 117DPS model had higher expression levels of *LAG3* and *PDCD1*, suggesting that these patients may benefit from immunotherapy. However, our study was based on the retrospective analysis and was unable to perform the clinical examination of the 117DPS model in real-world practice. Further studies are needed to explore the treatment strategy for the 117DPS-high subgroup.

The mechanism contributing to the poor prognosis in the high-risk group of 117DPS model is still unknown and may be quite complex. Firstly, the 117DPS model was derived from the CD34^+^CD117^dim^ population, which was demonstrated to be drug-resistant to chemotherapy in the t (8;21) AML patients [3]. On the other hand, the immune infiltration analysis showed that the high-risk group had a dysregulated immune system. Further analysis of the immune cells, such as the function state of T cells may help reveal the underlying mechanism.

In conclusion, we constructed a novel gene signature model in AML based on our previous findings [3, 4]. CD34^+^CD117^dim^ population signature, which may serve as a novel and accurate model, could predict the overall survival of patients with AML.

## Supplementary Information

**Additional file 1: Figure S1.** PPI construction based on the CD34^+^CD117^dim^ population signature (117DPS). Minimum required interaction score of 0.400 with disconnected nodes in the network hidden. Line colors indicated the type of interaction evidence.

**Additional file 2: Figure S2.** Forest plot of significantly prognostic genes through uni-variate Cox regression analysis in the training cohort. HR, hazard ratio; CI, confidence interval.

**Additional file 3: Figure S3.** Kaplan–Meier plot showing the survival differences of the six-gene in the 117DPS model for the GSE37642-GPL96 (*n* = 417) cohort.

**Additional file 4: Figure S4.** Sensitivity and specificity of the 117DPS model by a receiver operating characteristic (ROC) analysis of the validation cohorts (A, GSE37642-GPL570), (B, GSE12417-GPL96), (C, GSE12417-GPL570) and (D, GSE106291). AUC represents area under the curve.

**Additional file 5: Figure S5.** Gene set enrichment analysis (GSEA) of the high-risk groups (A) and low-risk groups (B) classified based on the 117DPS model in the GSE37642-GPL96 dataset.

**Additional file 6: Figure S6.** Analysis of the proportion of infiltrating immune cells in the low- and high- risk group of 117DPS model in the training cohort. Statistical significance was determined using two-sided Wilcoxon test.

**Additional file 7: Figure S7.** Analysis of the immune gene marker (*LAG3* and *PDCD1*) in the high- and low-risk group of 117DPS model in TCGA AML cohort (A) and Beat AML (B). * *P* < 0.05; ** *P* < 0.01; *** *P* < 0.001; Statistical significance was determined using two-sided Student's *t* test.

**Additional file 8: Table S1.** Highly expressed genes identified in CD34^+^CD117^dim^%-high group compared with CD34^+^CD117^dim^%-low group from OEP000629 of the National Omics Data Encyclopedia (NODE).

**Additional file 9: Tables S2–S7.** The distribution of clinical characteristics in the training cohort and validation cohorts. (GSE37642-GPL96, Table S2), (GSE37642-GPL570, Table S3), (GSE12417-GPL96, Table S4), (GSE12417-GPL570, Table S5), (Beat AML, Table S6) and (TCGA-LAML, Table S7).

## Data Availability

RNA-seq data of t (8;21) AML patients were deposited at the National Omics Data Encyclopedia (NODE) (http://www.biosino.org/node/project/detail/OEP000629). GEO datasets and RNA-seq data of Beat AML and TCGA LAML could be downloaded online.
