# Supplementary figures and images for: Integration of CD34+CD117dim population signature improves the prognosis prediction of acute myeloid leukemia
Source: J Transl Med. 2022 Aug 12;20:359. doi: 10.1186/s12967-022-03556-8 (PMC9373712; doi:10.1186/s12967-022-03556-8)

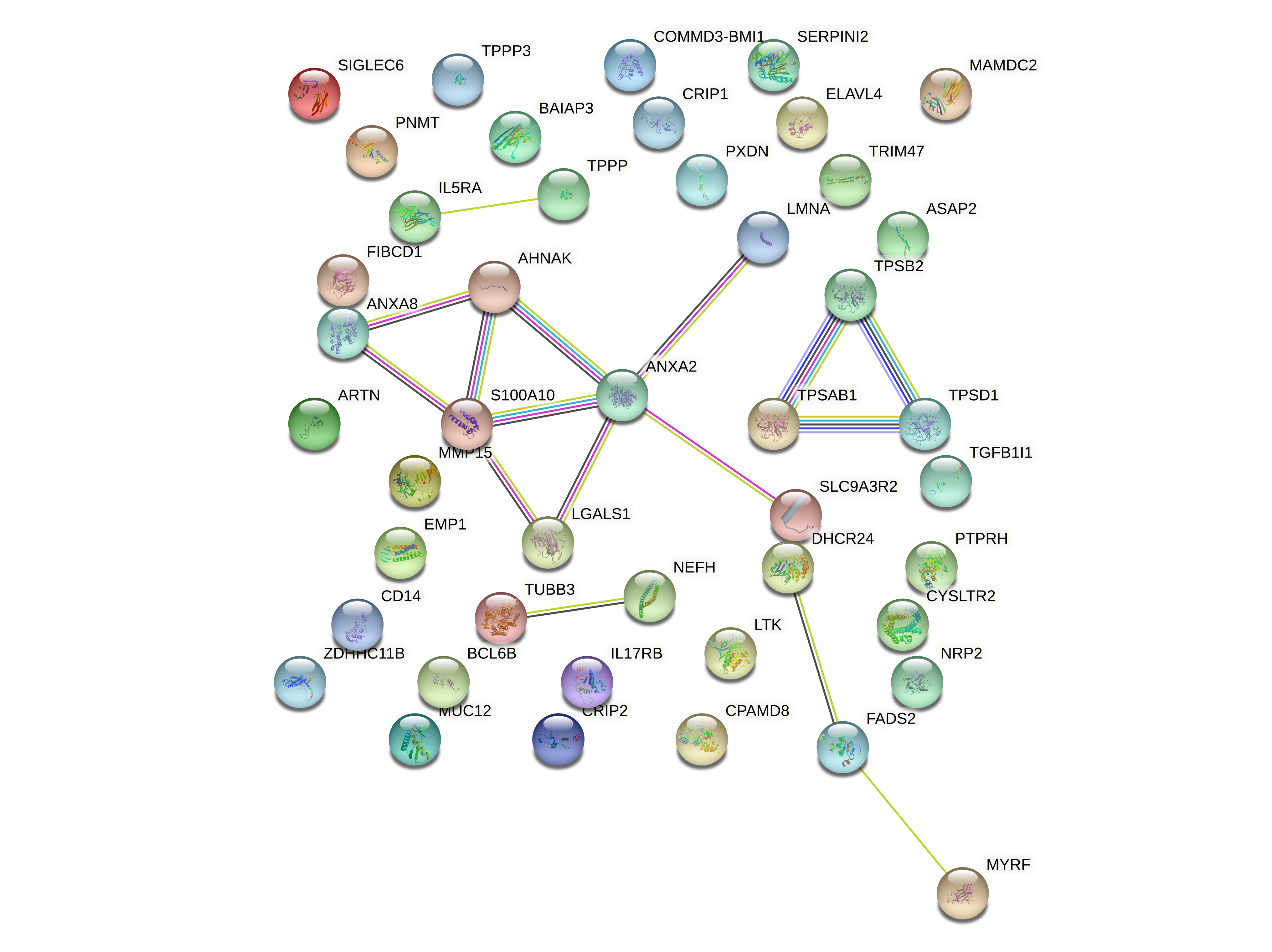

Supplement: Supplementary file 1 — Additional file 1: Figure S1. PPI construction based on the CD34+CD117dim population signature (117DPS). Minimum required interaction score of 0.400 with disconnected nodes in the network hidden. Line colors indicated the type of interaction evidence. [file 12967_2022_3556_MOESM1_ESM.tif]

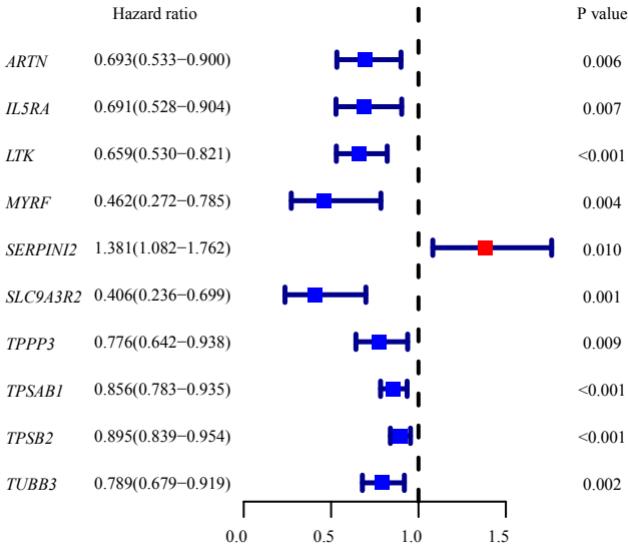

Supplement: Supplementary file 2 — Additional file 2: Figure S2. Forest plot of significantly prognostic genes through uni-variate Cox regression analysis in the training cohort. HR, hazard ratio; CI, confidence interval. [file 12967_2022_3556_MOESM2_ESM.pdf]

**ARTN**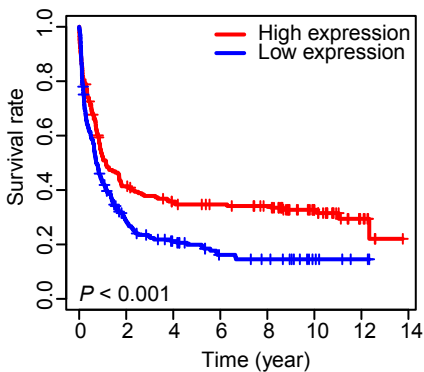**IL5RA**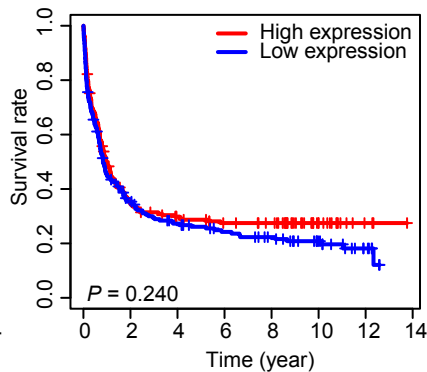**LTK**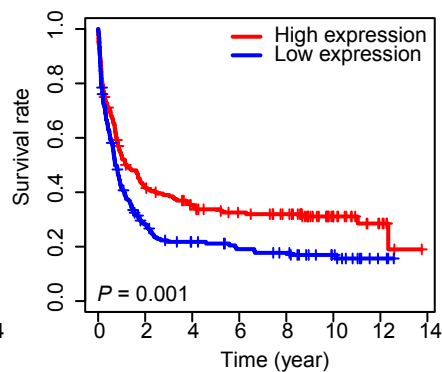**SERPINI2**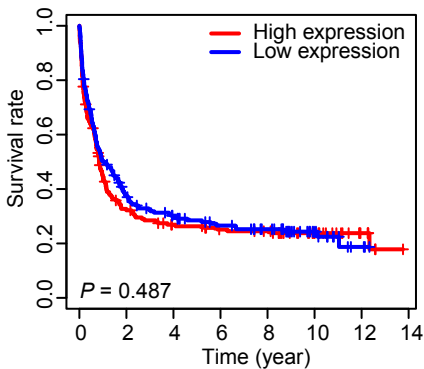**SLC9A3R2**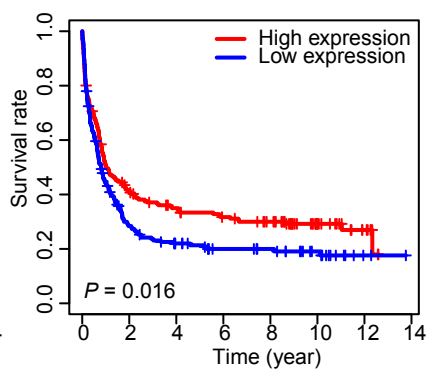**TPPP3**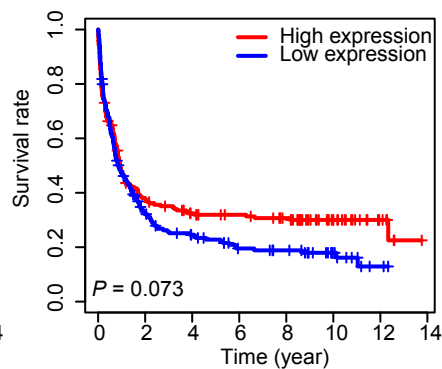

Supplement: Supplementary file 3 — Additional file 3: Figure S3. Kaplan–Meier plot showing the survival differences of the six-gene in the 117DPS model for the GSE37642-GPL96 (n = 417) cohort. [file 12967_2022_3556_MOESM3_ESM.pdf]

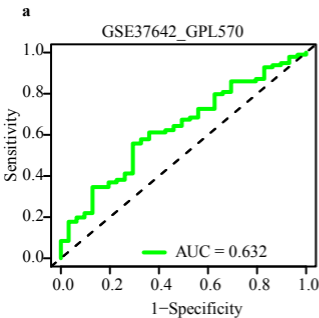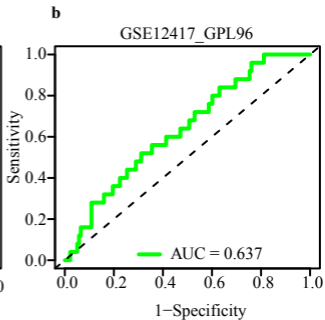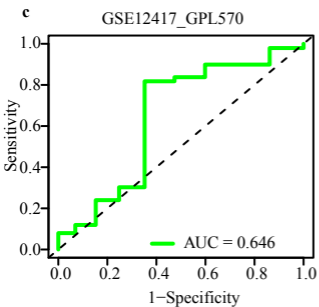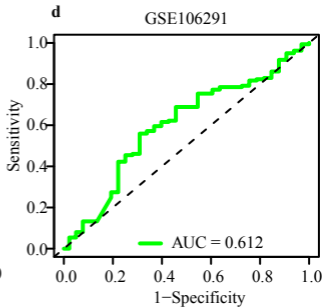

Supplement: Supplementary file 4 — Additional file 4: Figure S4. Sensitivity and specificity of the 117DPS model by a receiver operating characteristic (ROC) analysis of the validation cohorts (A, GSE37642-GPL570), (B, GSE12417-GPL96), (C, GSE12417-GPL570) and (D, GSE106291). AUC represents area under the curve. [file 12967_2022_3556_MOESM4_ESM.pdf]

**a**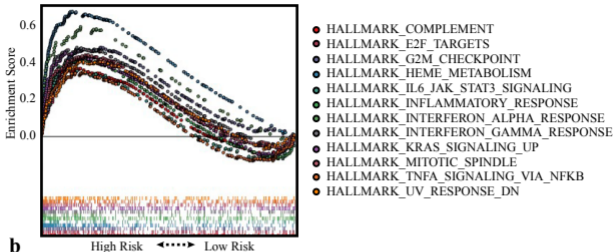**b**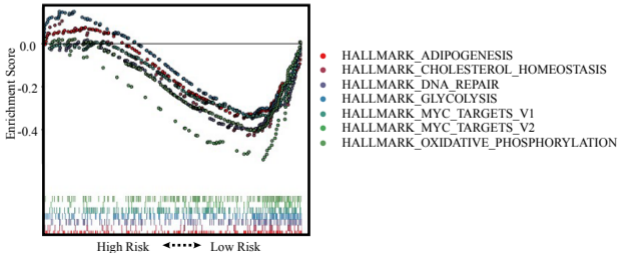

Supplement: Supplementary file 5 — Additional file 5: Figure S5. Gene set enrichment analysis (GSEA) of the high-risk groups (A) and low-risk groups (B) classified based on the 117DPS model in the GSE37642-GPL96 dataset. [file 12967_2022_3556_MOESM5_ESM.pdf]

**a**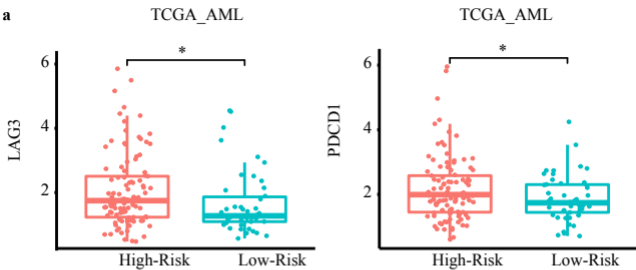**b**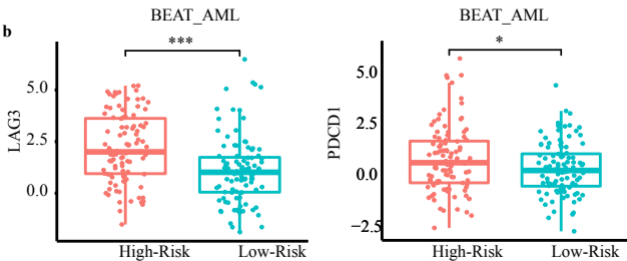

Supplement: Supplementary file 7 — Additional file 7: Figure S7. Analysis of the immune gene marker (LAG3 and PDCD1) in the high- and low-risk group of 117DPS model in TCGA AML cohort (A) and Beat AML (B). * P < 0.05; ** P < 0.01; *** P < 0.001; Statistical significance was determined using two-sided Student's t test. [file 12967_2022_3556_MOESM7_ESM.pdf]
