# Supplementary material for: Integration of CD34+CD117dim population signature improves the prognosis prediction of acute myeloid leukemia
Source: J Transl Med. 2022 Aug 12;20:359. doi: 10.1186/s12967-022-03556-8 (PMC9373712; doi:10.1186/s12967-022-03556-8)
Supplement: Supplementary file 6 — Additional file 6: Figure S6. Analysis of the proportion of infiltrating immune cells in the low- and high- risk group of 117DPS model in the training cohort. Statistical significance was determined using two-sided Wilcoxon test. [file 12967_2022_3556_MOESM6_ESM.pdf]

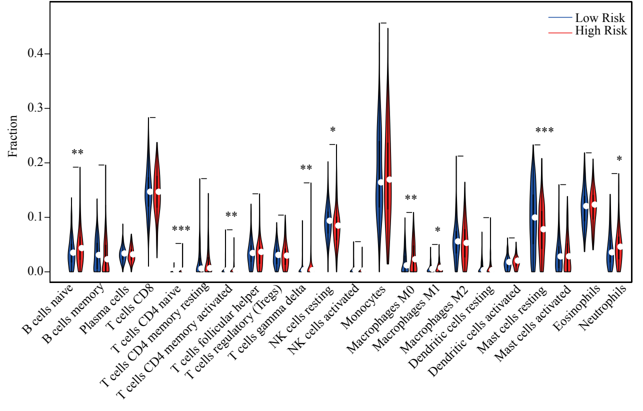

Low Risk
High Risk
Fraction
0.0
0.1
0.2
0.3
0.4
**
***
**
**
*
**
*
***
*
B cells naive
B cells memory
Plasma cells
T cells CD8
T cells CD4 naive
T cells CD4 memory resting
T cells CD4 memory activated
T cells follicular helper
T cells regulatory (Tregs)
T cells gamma delta
NK cells resting
NK cells activated
Monocytes
Macrophages M0
Macrophages M1
Macrophages M2
Dendritic cells resting
Dendritic cells activated
Mast cells resting
Mast cells activated
Eosinophils
Neutrophils
